# Percutaneous Coronary Intervention in Chronic Total Occlusion

**DOI:** 10.5935/abc.20180077

**Published:** 2018-05

**Authors:** Luiz Fernando Ybarra, Marcelo J. C. Cantarelli, Viviana M. G. Lemke, Alexandre Schaan de Quadros

**Affiliations:** 1 McGill University Health Centre, Montreal - Canadá; 2 Sociedade Brasileira de Hemodinâmica e Cardiologia Intervencionista, São Paulo, SP - Brazil; 3 Hospitais Leforte, São Paulo, SP - Brazil; 4 Hospital das Nações, Curitiba, PR - Brazil; 5 Hospital do Rocio, Campo Largo, PR - Brazil; 6 Instituto de Cardiologia / Fundação Universitária de Cardiologia - IC/FUC, Porto Alegre, RS - Brazil

**Keywords:** Coronary Artery Disease / complications, Coronary Occlusion, Percutaneous Coronary Intervention

## Abstract

Percutaneous coronary intervention in chronic total occlusion is a rapidly evolving area, being considered the last frontier of interventional cardiology. In recent years, the development of new techniques and equipment, as well as the training of specialized personnel, increased their success rates, making it the most predictable procedure available. Although the number of randomized and controlled studies is still limited, results from large multicentered registries allow us to safely offer this intervention to patients, as another treatment option along with the optimized drug treatment and myocardial revascularization surgery. This review summarizes the last and most relevant publications in the subject in order to provide an overall view of the field’s current status.

## Introduction

Percutaneous coronary intervention (PCI) in chronic total occlusion (CTO) has expressed great expansion and evolution with the development of new techniques and equipment, as well as with the training of specialized personnel. These factors have significantly raised success rates, making these procedures more effective and predictable. The aim of this manuscript is to present an update regarding indications, the aspects of the procedure, their results and clinical applicability of PCIs in CTO.

### Definition and epidemiology

CTO are defined as coronary obstructions which produce total occlusion of vessel lumen with TIMI 0 flow and duration longer than 3 months. Occlusions with minimal passage of contrast without opacification of the distal vessel are considered “functional CTO”.

CTOs are present in 18-52% of patients submitted to coronary angiography and who have coronary heart disease.^1^^-^^3^ More recent registries showed a prevalence between 16 and 20%.^4^^,^^5^ In these studies, the percentage of patients with CTO submitted to PCI was low. In two Canadian studies, only 9-10% of patients underwent PCI, while 57 to 64% of them remained in clinical treatment and 26 to 34% were referred for surgery.^2^^,^^4^

### Histopathological aspects

Understanding the histopathology of CTOs is an essential step to define the best percutaneous therapeutic strategy. The CTOs are consisted of a proximal and a distal cap, with an occluded segment between them. Histological analysis of these lesions showed that, in the proximal cap, more fibrous and calcified components are more predominant than in the distal one and which, despite complete angiographic occlusion, may have intravascular microchannels which cross the occluded segment.^6^^-^^8^ Blunt caps present histopathological differences when compared to tapered ones, with less frequent intravascular microchannels.^7^

The viability of the myocardium irrigated by the occluded artery is maintained by collateral circulation, which may be developed by angiogenesis or by the action of circulating endothelial progenitor cells.^9^ It is difficult to assess the ability of collateral vessels to maintain coronary perfusion, and the angiography is not the most accurate method to predict the functionality of collaterals. The traditional knowledge that the occluded vessel has ‘adequate and sufficient collaterals’ for CTO ischemia prevention is challenged by physiological evidence with fractional flow reserve (FFR) analysis.^10^

### Selection of patients

European guidelines for myocardial revascularization recommend that CTO PCI should be considered for ischemia reduction in the corresponding myocardial territory and/or for reduction of angina (class IIa, level of evidence B).^11^ According to the guidelines for the management of stable coronary disease, indications for CTO revascularization should be the same as one for a subtotal stenosis, provided that viability, ischemia of a sufficiently large territory and/or angina symptoms are present.^12^ With the current techniques, equipment, success and complication rate, patient selection should not depend on the type of lesion (total, subtotal or severely obstructive), but rather on symptoms and on the findings in complementary tests.^13^ Although it is essential to ensure the viability of the myocardial territory supplied by a chronically occluded vessel, the presence of collateral circulation does not prevent the occurrence of ischemia in this area.^10^ Thus, the size of the collateral circulation should not be used as a criterion to contraindicate revascularization.

### Ischemia and myocardial viability

In addition to symptoms, evaluating the presence of ischemia and myocardial viability are fundamental steps. In asymptomatic patients, the evaluation of ischemia before CTO PCI is considered. The analysis of the receiver operating characteristic (ROC) curve of a cohort involving 301 patients showed 12.5% as the optimal amount of ischemia pre-procedure in order to identify patients who have benefited from the intervention in terms of ischemia reduction.^14^

The presence of myocardial viability is important to identify patients who would benefit from CTO recanalization. A combination of viability parameters may predict better and more accurate myocardial function than the use of a single parameter, such as transmural extension of the infarction, evaluation of the contractile reserve with dobutamine and thickening of the normal myocardial wall in cardiac magnetic resonance, especially in segments with intermediate extension of infarction.^15^

### The procedure

#### Planning the procedure

**Figure 1:**
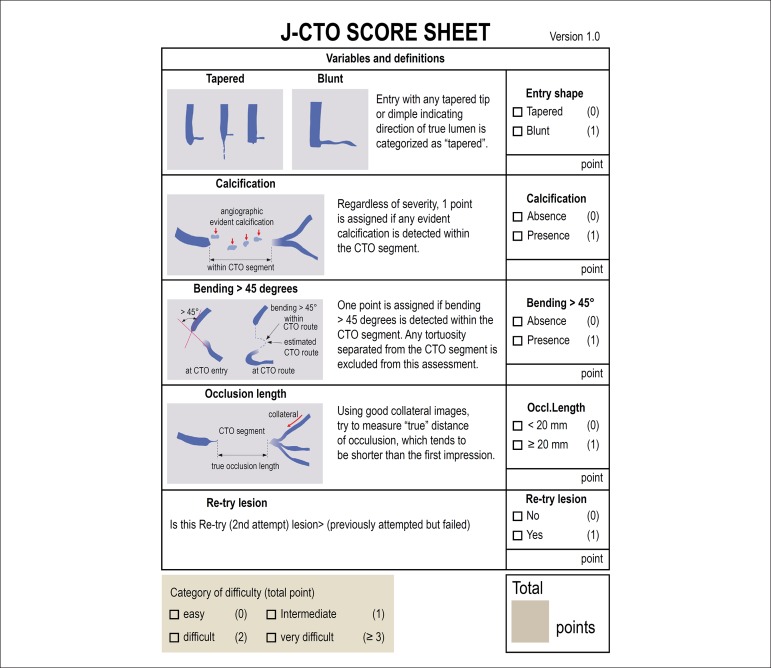
J-CTO score: angiographic score used to estimate the probability of success of the procedure. Five variables were analyzed: proximal cap (tapered or blunt), presence of calcification in chronic total coronary occlusions (CTO), presence of angulation greater than 45 degrees within the CTO segment, length of occlusion (greater or equal to 20 mm) and unsuccessful previous approach attempt. The degree of difficulty of the procedure increases the greater the J-CTO score.

The use of angiographic scores to estimate the probability of success and the type of approach is essential in the planning of the procedure. The J-CTO score is the oldest and most widespread one (Figure 1).^16^ Patients with higher J-CTO scores have significantly lower success rates, longer procedures, greater use of contrast, and more frequent use of the retrograde approach.^17^^,^^18^ Other relevant scores are the PROGRESS-CTO score and the Clinical and Lesion (CL) score.^19^^,^^20^ These three scoring systems present similar predictive abilities for technical success, being more accurate in anterograde cases.^21^

#### Overall technical aspects

The performance of *ad hoc* CTO PCI to the diagnostic procedure is widely discouraged, in order to allow a careful and appropriate review of angiography, obtaining informed consent and limiting the use of contrast and procedure.

Contrast injection in the occlusion vessel simultaneously with injection into the donor vessel of the collateral circulation (simultaneous contralateral injection) is indispensable for the determination of CTO characteristics, including lesion length, proximal and distal cap morphology, lateral branches and the extension and morphology of the collateral branches. Anterograde injection should be avoided from the moment that subintimal dissection occurs in the anterograde space, since the hydraulic pressure of the contrast injection may increase the dissection plane, increasing the subintimal bruise. The use of combinations of bi-femoral, femoral-radial or bi-radial accesses will depend on the staff's preference, the availability of the necessary materials, the patient's characteristics, the procedure and the anatomy.^22^

In order to have better planning of treatments of CTOs, the so-called hybrid algorithm has been developed, which has allowed to maximize success and reduce the time of the procedure, radiation and the use of contrast, enabling the teaching and dissemination of techniques and reducing inter-and intra-operator approach variability and success rates. The core of this algorithm is the rapid identification of the failure of each strategy followed by immediate exchange for another type of technique.

**Figure 2:**
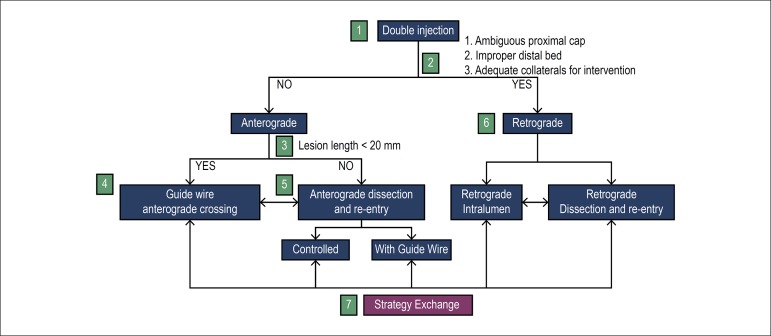
Hybrid Algorithm for Crossing Chronic Coronary Occlusions: The hybrid algorithm begins with double coronary injection (Item 1), which allows the evaluation of several angiographic parameters (Item 2) and selection of the type of primary approach: anterograde (Items 3 to 5) or retrograde (Item 6). Changes in strategy are performed (Item 7) depending on the evolution and progress of the procedure.

The algorithm or hybrid approach consists of two paths (anterograde and retrograde) and two ways of crossing CTO: through true lumen or through the subintimal space (with dissection and then re-entry to the true lumen). The definition of which path to use and how to cross the occlusion is determined by 4 main anatomical factors: proximal cap anatomy, occlusion length, presence of a disease-free zone for reentry in the distal vessel and presence of usable septal or epicardial collaterals (Figure 2).

Even by using modern techniques in centers of excellence, failure can still occur, which does not make a new attempt unfeasible.^23^ Unsuccessful cases in which the lesion is “modified” - especially the proximal cap, whether with multiple dissections made by wire-specific guides or micro catheters, whether through balloon angioplasty or even subintimal approach - are called “investment procedures”, which aim to facilitate a future attempt at recanalization.^23^

#### Anterograde technique with wire scaling

Staggering of anterograde wires is the most commonly used approach. A micro catheter is advanced to the proximal cap, followed by attempts to cross CTO using specific guidewires according to the morphology of the cap. Generally, it starts with a soft and fine-tipped guidewire (1.0 g), coated with polymer. If the crossing is unsuccessful, a slightly heavier wire (4.0 g), also polymer coated, or a sharp, tapered 12-gauge wire is used. The recent introduction of rigid composite core guidewires seems to further enhance the success of anterograde crossover by allowing better torque control and transmission.

Understanding the guidewire path is critical both to increase the likelihood of success and to minimize the risk of complications. If the guidewire enters the true distal lumen (confirmed in two orthogonal projections), the micro catheter is advanced through the occlusion and the guidewire is replaced by a traditional one, followed by balloon angioplasty and stent implantation. If the guidewire comes out of the vessel architecture, it must be retracted and redirected. If the guidewire crosses the occlusion but enters the subintimal space, reentry into the true lumen can be achieved by the “parallel wires” technique (less commonly used today) or the use of a dedicated re-entry system.

#### Anterograde dissection and reentry technique

Dissection and reentry are related to the intentional use of the subintimal space to cross the occlusion, a strategy that should be considered when the CTO extension is greater than 20 mm. Strategies to induce limited and controlled dissections seem to have better short- and long-term results when compared to those that cause extensive dissections.^24^^-^^26^

Controlled dissection can be achieved with dedicated micro catheters that create a limited dissection plane. The reentry is obtained with the help of a specific balloon for this purpose. A recent study demonstrated that the use of dedicated equipment was associated with lower rates of major cardiovascular events (MACE) (4.3 *vs*. 15.4%, p = 0.02) and target vessel revascularization (3.1 *vs*. 15.5%, p = 0.02) when compared to older techniques.^27^

#### Retrograde technique

The retrograde approach to CTO crossing can significantly increase success rates, particularly in more complex lesions. It is considered the first line strategy when the proximal cap is ambiguous, the antegrade reentry zone is not adequate or the distal cap ends at a bifurcation. Retrograde crossing by grafts (especially venous grafts) and by septal collaterals are preferred to epicardial collaterals because they are easier to traverse and present lower risk of tamponade in case of perforation or rupture.^28^^,^^29^ Through a collateral, the guidewire proceeds to the distal region of the occlusion and, from this point, the CTO is crossed in the opposite direction to the blood flow.^30^ Retrograde crossing by the true lumen is generally easier, once that the distal lumen tends to have more favorable (softer, pencil-like, less ambiguous) characteristics than the proximal one.^8^ If true lumen crossing is not possible, dissection and re-entry techniques, other than anterograde techniques, may also be applied.

#### Choice of *stents*

Intra-stent restenosis after CTO PCI with conventional stents was of approximately 50%, which practically prevented its use in this scenario. With the drug-eluting stent implantation, clinical outcomes improved significantly, leading to lower restenosis rates (relative risk: 0.25, 95% CI: 0.16-0.41, p < 0.001), reocclusion (relative risk: 0.30, 95% CI: 0.18-0.49, p < 0.001) and new target vessel revascularization (relative risk: 0.40, 95% CI: 0.28-0.58, p < 0.001).^31^^-^^34^ Thus, the use of drug-eluting stents became mandatory.

The use of absorbable vascular platforms for the treatment of CTO has been evaluated in a number of studies, with promising results.^35^^-^^38^ However, following the long-term results of the ABSORB III study indicating an increase in the rates of very late thrombosis, its use will probably be restricted.^39^

#### Use of intravascular imaging methods

Two intravascular imaging methods are currently available for clinical use: intravascular ultrasound (IVUS) and optical coherence tomography. Optical coherence tomography requires a fluid injection (usually contrast) to be performed, which may lead to an increase of an existing dissection plane, and therefore is not usually used in CTO ICPs.

The IVUS, on the other hand, can be used in a variety of procedure situations (defining the ambiguity of the proximal cap, facilitating the re-entry into the true lumen, limiting the dissection plane and confirming the distal positioning of the guidewire in the true lumen), in addition to those in which it is used in traditional PCIs.^40^^-^^43^

#### Results and complications

The hybrid approach has allowed success rates of 85-90% in the most recent studies.^23^^,^^44^^-^^47^ The occurrence of in-hospital MACE ranges from 0.5 to 2.6%.^24^^-^^27^ However, these procedures are still at larger risk of complications when compared to PCIs of non-CTO lesions.^48^

The incidence of peri-procedural myocardial infarction (MI) is associated with factors such as retrograde technique, moderate/severe calcification, and unsuccessful procedures.^49^^,^^50^ The impact of MI peri-procedure on mid- and long-term follow-up is still not well defined.^51^^,^^52^

The prevalence of bifurcation lesions in CTO interventions is 33%. The lateral branches should be considered and treated as in conventional intervention procedures.^53^ The occlusion of lateral branches may affect the long and short-term outcomes of CTO PCI, being more frequent when the stent is implanted on the branch and when the technique of dissection and reentry is used.^54^

CTO ICPs are at higher risk of perforations than those in non-occlusive lesions. In centers of excellence, using contemporary treatment, the incidence of perforations is approximately 1-2%.^55^ The management of this complication varies with the type of perforation, and the operator should be familiar with the techniques and devices necessary for the treatment.^56^

The high doses of radiation required to perform increasingly complex procedures are of concern to physicians and patients. Protocols dedicated to CTO interventions, more modern equipment and the adoption by operators of attitudes that reduce exposure to ionizing radiation have allowed these procedures to be carried out with increasingly smaller radiation doses.^57^^,^^58^

The decision to interrupt the procedure should be individualized, and there is no scientific evidence to support the use of specific criteria. Five parameters are usually used (radiation, contrast, complications, futility and risk/benefit ratio), but the final decision depends heavily on the judgment of the operator.

Intra- and post-hospital care should be the same as any other complex PCI, taking into account the complications that occurred during the procedure and the amounts of contrast and radiation used.

#### Clinical benefits

Successful CTO recanalization is associated with a number of clinical benefits, such as improved angina, quality of life and physical limitation, improved ventricular function, and decreased mortality when compared to patients whose recanalization was not successful.

Sapontis et al. evaluated the quality of life of 1,000 patients submitted to OCT PCI. One month follow-up showed a significant improvement in all domains of the Seattle Angina Questionnaire (SAQ), Rose Dyspnea Scale and PHQ-8 scores.^47^ In another study with 184 patients in a one-year follow-up, a significant improvement in the quality of life of patients submitted to successful CTO PCI was also observed. The improvement was similar in all patients, regardless of their clinical, anatomical or procedural complexity.^59^ In Mashayekhie et al., evaluated the impact of CTO recanalization on the physical capacity of 50 patients undergoing cardiopulmonary testing before and after 7 months. The successful intervention improved exercise capacity (maximal oxygen consumption and anaerobic threshold increased by 12 and 28%, respectively; p = 0.001 for both).^60^

Several observational studies show a relationship of CTO recanalization in the reduction of clinical events. Jang et al. compared CTO revascularization (by PCI or by surgery) with drug therapy in 738 patients with well-developed collaterals. The combined prognostic analysis at 42 months showed a 73% reduction in the incidence of cardiac death.^61^ The Italian CTO Registry assessed the clinical outcomes of 1,777 patients, showing lower cardiac mortality (1.4, 4.7 and 6.3%, p < 0.001) and MACE at one year (2.6, 8.2 and 6.9%, p < 0.001) in patients treated with PCI when compared to clinical treatment or surgery. In this study, the group receiving optimized medical treatment presented higher rates of MACE, death and re-hospitalization.^62^

To date, three randomized controlled trials have evaluated the potential benefits of CTO PCI. The EXPLORE study included 304 patients with acute myocardial infarction (AMI) who underwent primary PCI and presented CTO in a non-infarct-related artery. They were randomized to CTO PCI in a second moment *versus* optimized medical treatment (OMT). At the 4-month follow-up, similar left ventricular function was observed in both groups, although a significant improvement in the ejection fraction was observed in the subgroup of patients with anterior wall AMI. The inclusion of patients without viability research may have limited a possible PCI positive result.^63^

The DECISION-CTO study randomized 834 patients with CTO for OMT *vs*. OMT + CTO PCI.^64^ In the 3-year clinical follow-up, CTO PCI as the initial treatment strategy did not provide a decrease in MACE, the primary outcome of the study. However, this study had important limitations: it was terminated early before reaching the pre-specified number of patients required, with low inclusion rate of patients per center; patients with low severity and low symptomatic status were included; and there was high cross-over rate for the intervention group (20%).

The Euro CTO Trial randomized 407 patients with stable coronary disease for OMT *vs*. OMT + CTO PCI. The primary outcome was an improvement in the quality of life, as assessed by SAQ.^55^ Although there were also limitations related to selection bias (termination of the study with only one third of the planned sample due to slow inclusion), randomized patients to PCI CTO showed a significant improvement in angina frequency, physical limitation, and quality of life in the 12-month follow-up.

In a recent meta-analysis including 9 studies with more than 6,400 patients, the long-term clinical outcomes of successful CTO recanalization were compared to those in whom the recanalization was unsuccessful. In this study, the risk of death, AMI and MACE was approximately 50% lower in patients with CTO recanalization, with a 90% lower incidence of myocardial revascularization.^65^

#### Brazilian reality

The percutaneous treatment of CTO in Brazil with the contemporary techniques described here can still be considered incipient due to the limited availability of dedicated materials in our country, affecting the adequate training of the operators. Recently, following the worldwide trend of treatment of these lesions based not only on the anatomy, but also on the symptoms and the clinical indication, several institutions and interventionists started to dedicate themselves to this area. The Brazilian Society of Hemodynamics and Interventional Cardiology (SBHCI) has stimulated this development, having already organized two dedicated courses (CTO Summit Brazil 2016 and 2017) and supporting specific regional events.

The role of specific training to perform this type of procedure is imperative, both for the knowledge of the techniques and the equipment used. Most operators develop their skills by participating in courses and procedures with proctors. There are also dedicated training programs, however limited to few centers in the world.^48^^,^^66^

## Conclusion

The CTO PCI is a rapidly advancing field. With the use of the right equipment and current techniques, high volume and expertise centers achieve high success rates. Although current evidence is favorable to PCI, prospective randomized controlled good quality trials are still needed to define the best indications and the most appropriate techniques for intervention in this challenging population.
